# Palliative care in the home: a scoping review of study quality, primary outcomes, and thematic component analysis

**DOI:** 10.1186/s12904-018-0299-z

**Published:** 2018-03-07

**Authors:** Mark Hofmeister, Ally Memedovich, Laura E. Dowsett, Laura Sevick, Tamara McCarron, Eldon Spackman, Tania Stafinski, Devidas Menon, Tom Noseworthy, Fiona Clement

**Affiliations:** 1 Department of Community Health Sciences, University of Calgary, Teaching Research and Wellness Building, 3280 Hospital Drive NW, Calgary, Alberta T2N 4N1 Canada; 2 O’Brien Institute for Public Health, Health Technology Assessment Unit, Teaching Research and Wellness Building, 3280 Hospital Drive NW, Calgary, Alberta T2N 4N1 Canada; 3 School of Public Health, University of Alberta, 3-300 Edmonton Clinic Health Academy, 11405-87 Ave, Edmonton, Alberta T6G 1C9 Canada

## Abstract

**Background:**

The aim of palliative care is to improve the quality of life of patients and families through the prevention and relief of suffering. Frequently, patients may choose to receive palliative care in the home. The objective of this paper is to summarize the quality and primary outcomes measured within the palliative care in the home literature. This will synthesize the current state of the literature and inform future work.

**Methods:**

A scoping review was completed using PRISMA guidelines. PubMed, Embase, CINAHL, Web of Science, Cochrane Library, EconLit, PsycINFO, Centre for Reviews and Dissemination, Database of Abstracts of Reviews of Effects, and National Health Service Economic Evaluation Database were searched from inception to August 2016. Inclusion criteria included: 1) care was provided in the “home of the patient” as defined by the study, 2) outcomes were reported, and 3) reported original data. Thematic component analysis was completed to categorize interventions.

**Results:**

Fifty-three studies formed the final data set. The literature varied extensively. Five themes were identified: accessibility of healthcare, caregiver support, individualized patient centered care, multidisciplinary care provision, and quality improvement. Primary outcomes were resource use, symptom burden, quality of life, satisfaction, caregiver distress, place of death, cost analysis, or described experiences. The majority of studies were of moderate or unclear quality.

**Conclusions:**

There is robust literature of varying quality, assessing different components of palliative care in the home interventions, and measuring different outcomes. To be meaningful to patients, these interventions need to be consistently evaluated with outcomes that matter to patients. Future research could focus on reaching a consensus for outcomes to evaluate palliative care in the home interventions.

**Electronic supplementary material:**

The online version of this article (10.1186/s12904-018-0299-z) contains supplementary material, which is available to authorized users.

## Background

By 2056, 480,000 Canadian deaths per year are predicted with 90% of those deaths being eligible for palliative care [1]. The aim of palliative care is to improve the quality of life of patients and families through the prevention and relief of suffering [2]. Frequently, patients may choose to receive palliative care in the home. Palliative care in the home is the provision of specialized palliative care in the patient’s home, most often provided by nurses and/or physicians with or without connection to a hospital or hospice [3]. Three factors have been identified as contributing to the decision to receive palliative care in the home: fulfilling a promise to provide care to the patient at home, the wish to maintain a ‘normal family life’, and previous negative experiences in institutional care settings [4].

In a recent survey of 1200 Canadians, greater than 70% of respondents preferred to be at home near death [5]. Palliative care in the home interventions vary widely, from interventions attempting to provide hope, to informal caregiver advising or after-hours night respite [6–8]. Frequently, palliative care is evaluated retrospectively through proxy reports and routine data collection [9]. Palliative care has also been evaluated in terms of cost and resource utilization [10]. Although location of death, at home or in hospital, has frequently been used as a metric to evaluate palliative care in the home, this measure is widely criticized as it accounts for only the conclusion to the process of dying [11].

Further, in 2013, a Cochrane review of home based palliative care for patients and their caregivers found positive effects on symptom burden, and no impact on caregiver grief, compared to usual care [10]. Another recent systematic review found that regular communication with medical professionals, spiritual needs, mobility assistance and financial support, information about illness progression, and respite options for caregivers were not adequately addressed by palliative care in the home [12].

The breadth of the body of literature examining palliative care in the home is unknown. Palliative care in the home interventions vary significantly in terms of the components of care, providers offering the interventions, and target recipients. The objective of this scoping review is to clarify the current state of the palliative care in the home literature in terms of study quality, primary outcomes measured, and begin to categorize palliative home care interventions. Summarizing the current literature will also assist future contributions to develop intervention evaluations that facilitate comparison and identify value for stakeholders.

## Methods

### Search strategy

A scoping review was completed [13]. Quality of included studies was assessed to increase the utility of this scoping review and to add the needed quality lens to the literature. In August 2016, the following electronic databases were searched from inception: PubMed, Embase, Cumulative Index to Nursing and Allied Health Literature (CINAHL), Web of Science, Cochrane Library, EconLit, PsycINFO, Centre for Reviews and Dissemination, Database of Abstracts of Reviews of Effects, and National Health Service Economic Evaluation Database. Palliative care in the home experts were contacted to identify additional papers. Search results were limited to those published after the year 2000, to ensure that included studies were representative of modern palliative home care. Only published studies were reviewed thus ethics approval was not required. Grey literature was not included. Only English search results with human subjects were included in abstract review. Preferred reporting items for systematic reviews and meta-analyses (PRISMA) guidelines were followed to ensure methodological best practices [14].

The search strategy consisted of three concepts. First, terms for palliative care such as “palliative care,” “terminally ill,” and “end of life care” were searched. Second, terms for home care such as “home care services/trends,” “health system pathway,” and “component” were searched. Third, terms for outcomes such as “health care quality, access, and evaluation,” “quality,” and “patient satisfaction” were searched. These three concepts were combined using the Boolean operator “and.” The PubMed search strategy is included in Additional file 1: Appendix 1.

### Study selection

Titles and abstracts were reviewed for eligibility in duplicate. Inclusion criteria were: 1) must assess at least one component of palliative care; 2) must report location of care as “home of patient” irrespective of if care was delivered in an individual home, hospice, or continuing care; 3) report on any outcome; and 4) report primary data. No study designs were excluded. Abstracts included by either reviewer proceeded to full text review. At full text review, a third reviewer was consulted to resolve inclusion disagreement.

### Analysis

Included studies were classified by primary outcome. Data extracted, independently and verified by a second reviewer, included objective, methods, primary outcomes, and themes addressed. Study quality of randomized controlled trials was assessed using the Cochrane Risk of Bias (ROB) tool [15]. Controlled before and after studies was assessed using the same criteria as randomized controlled trials, but reported as high risk of bias on random sequence generation and allocation sequence concealment [16]. Quality of cohort studies was assessed with Newcastle-Ottawa criteria [17]. Quality of qualitative studies was assessed using the Critical Appraisal Skills Programme Qualitative Checklist, as recommended by Cochrane [18, 19]. Cross-sectional study quality was assessed using the Cochrane suggested “Quality Assessment Tool for Quantitative Studies” [20, 21]. Cost studies did not include outcomes in analysis, and were therefore not appropriate for any existing validated quality assessment tools.

Thematic component analysis was used to describe palliative home care interventions [22]. Face validity of intervention themes was established with expert opinion. The five themes categorizing palliative home care interventions were: accessibility of healthcare, family and caregiver support, individualized patient centered care, multidisciplinary care provision, and quality improvement.

## Results

### Study characteristics

**Fig. 1:**
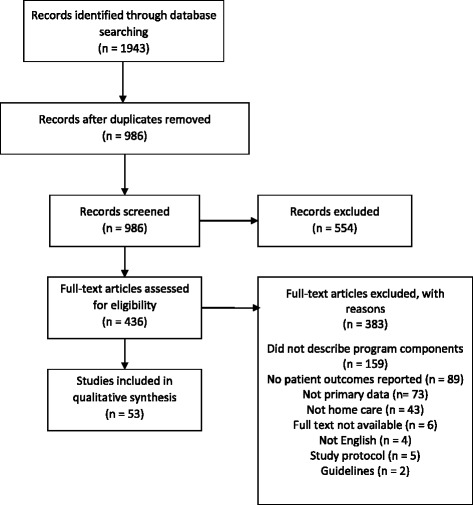
PRISMA flow diagram

Searches of electronic databases returned 1993 records (Fig. 1). Following removal of duplicates, 986 records remained for title/abstract screening. Five hundred four articles were excluded at the title/abstract screening stage. The full text of 436 articles were assessed for inclusion, and 53 articles were included in the final data set (Fig. 1). Qualitative study designs were most numerous with 10 studies identified [8, 23–31]. This was followed by retrospective cohort study designs, with eight studies included [32–39]. The country in which the greatest number of studies was conducted was the United States, with 12 studies [32, 33, 40–49]. This was followed by Canada with nine studies [6, 30, 35, 37, 38, 50–53]. Included study characteristics are presented in Additional file 2: Table S1.

### Quality

**Fig. 2:**
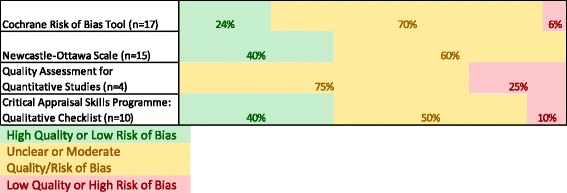
Quality assessment of included studies

Included studies were assessed with the scales indicated in Fig. 2, and a category for overall quality was added. When using the Cochrane Risk of Bias (ROB) tool, categories assigned low risk of bias were given a value of one, categories assigned moderate risk of bias were given a value of two, and categories assessed as high risk of bias were given a value of three [15]. The sum of all ROB values was calculated. Values from 6 to 10 were categorized as low risk of bias or high quality, values from 11 to 14 were categorized as unclear/moderate quality, and values from 15 to 18 were categorized as low quality with the Cochrane ROB tool. When using the Newcastle-Ottawa scale, from one to three stars was categorized as low quality, four to six stars was categorized as unclear/moderate quality, and seven to nine stars was categorized as high quality [17]. In qualitative studies assessed using the Critical Appraisal Skills Programme Qualitative Checklist, the sum of the number of times “yes” was used as the answer to questions was calculated [18]. From one to three “yes” answers was categorized as low quality, four to six “yes” answers was categorized as unclear/moderate quality, and seven to nine “yes” answers was categorized as high quality.

Most studies had an unclear risk of bias or quality (Fig. 2). The only study tool used that did not identify any studies with high quality was the quality assessment for quantitative studies tool.

### Outcomes identified

**Fig. 3:**
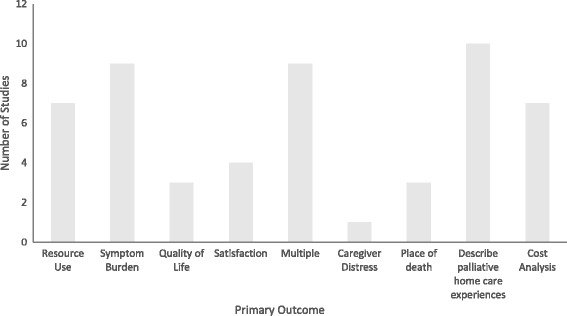
Primary outcomes of included studies

Multiple outcomes described studies in which the objective statement identified a combination of resource use, symptom burden, quality of life, satisfaction, caregiver distress, or place of death as the primary outcome (Fig. 3). The most commonly reported outcome was descriptive in nature with the objective of the study being to describe experiences with services offered.

### Types of components

Intervention components were analyzed thematically, and five themes were identified (Table 1). Study interventions were described with one or more themes. Theme one described interventions in which a major component was an increase in the ability of participants to access healthcare as required. Theme two, caregiver support, was identified in 12 studies and reported effects on caregivers or provided services to caregivers. Theme three, individualized patient centered care, was identified in 35 studies. Individualized patient centered care described interventions in which tailored care to participant needs. Theme four, multidisciplinary care provision, was identified in 24 studies and described interventions with care provided by disciplines other than, or in addition to, physicians and nurses. To be categorized as addressing the theme of quality improvement, the publication had to describe the palliative care in the home intervention as a change to previously existing palliative care in the home service with the goal of improvement.

**Table 1:** Overview of identified themes

Theme	Description	Examples	Included Studies within Theme (n (%))
Accessibility of Healthcare	Interventions in which a major component was increased ability of study participants to access healthcare when required.	24/7 on call care access Access to specialists	27 (50.9)
Caregiver Support	Interventions in which effects for both patients and caregivers were reported, or services are also provided to caregivers.	Patient and caregiver education Advice for informal caregivers	12 (22.6)
Individualized patient centered care	Interventions tailored to participant needs during delivery.	Care plan for symptom management Coordination of care	35 (66.0)
Multidisciplinary care provision	Interventions provided by healthcare disciplines other than general practice physicians and nurses.	Social work involvement Physiotherapist involvement	24 (45.3)
Quality improvement	Interventions described as changes to previously existing palliative care in the home services with the goal of improvement.	Tablet use for documentation Intervention standardization	6 (11.3)

## Discussion

Fifty-three studies of various study designs, reporting on diverse outcomes and of varying quality were identified within the palliative home care literature. Of note, there are gaps in knowledge related to the outcome of caregiver distress, and the theme of quality improvement of palliative care in the home interventions. The extensive and heterogeneous nature of this body of literature limits our ability to make generalizations about effective palliative care in the home.

Palliative care in the home interventions were evaluated with the primary outcomes of resource use, cost, or place of death in nearly one third of included studies. Some have argued that these outcomes are not sufficient to assess the effectiveness of the intervention [54, 55]. The effectiveness measure should link to the objective of the intervention; place of death may be a less appropriate outcome than the location of care preceding death [55]. Our findings underscore the need to expand outcome measurement beyond routinely collected data to measure outcomes that are more tightly linked to the objective of palliative care in the home.

Included interventions differed in terms of providers delivering care and intervention components. The theme of individualized patient centered care was present in 66% of included studies and highlights the focus of these interventions on meeting patient needs. However, the diverse nature of these interventions makes generalizations about what components contributed to positive outcomes difficult. In addition, nine different primary outcomes were identified in the included studies. This diversity in outcomes makes it difficult to understand which components are effective or not. Consensus about which outcomes would be required to demonstrate that an intervention is effective would significantly advance the developing body of literature.

Although quality was mixed, all intervention evaluations reported positive outcomes. This supports the intuitive hypothesis that palliative care in the home is good for patients. However, this may be due to publication bias. Heterogeneity, in terms of intervention components and measurement of primary outcomes, inhibit our ability to assess whether studies with negative effects remain unpublished.

Similar to a meta-ethnography exploring patient and caregiver priorities for palliative care in the home, a focus on the increased availability of healthcare providers, symptom relief, and including caregivers as recipients and participants in care were prominent in intervention evaluations [56]. Like the findings of this study, a systematic review of systematic reviews of palliative care models also highlighted the diversity present in this body of literature, and the lack of consensus regarding outcome measurement [57].

There are limitations to our findings. The search strategy used was broad, however there is the possibility that appropriate studies may have been missed. To mitigate this risk, experts were contacted to identify additional literature. During our initial scoping work, the identified grey literature was reported in enough detail to contribute to this work without accompanying interviews to provide additional details. Thus, grey literature was excluded. This may have biased our results as the field is moving rapidly and novel interventions may not be reported in the published literature yet. Lastly, the interventions described in included studies are complex and multifaceted. This may have led to misclassification errors in the thematic analysis. However, we anticipate this error being small as it was done in duplicate and consensus was reached for each classification.

To move this field forward, future research should attempt to reach consensus on meaningful outcomes to be evaluated for palliative care in the home. Clinical events, costs, and place of death are routinely captured, but these may not match patient and caregiver priorities. Ideally, this consensus process would include key stakeholders representing all facets of palliative care at home: patients, caregivers, clinicians, and decision-makers.

## Conclusions

There is variety in the quality of reporting, components of palliative care in the home interventions, and outcomes measured. To be meaningful to patients, these interventions need to be consistently evaluated with outcomes that matter to patients. Future research could focus on reaching a consensus for outcomes to evaluate palliative care in the home interventions.

## Additional files

Additional file 1: **Appendix 1.** PubMed search strategy. (DOCX 15 kb)

Additional file 2: **Table S1.** Included study characteristics. (DOCX 48 kb)

## Abbreviations

CINAHL: Cumulative index to nursing and allied health literature
PRISMA: Preferred reporting items for systematic reviews and meta-analyses
ROB: Risk of bias
