# Supplementary material for: Palliative care in the home: a scoping review of study quality, primary outcomes, and thematic component analysis
Source: BMC Palliat Care. 2018 Mar 7;17:41. doi: 10.1186/s12904-018-0299-z (PMC5842572; doi:10.1186/s12904-018-0299-z)
Supplement: Supplementary file 1 — Appendix 1. PubMed search strategy. (DOCX 15 kb) [file 12904_2018_299_MOESM1_ESM.docx]

**Additional file 1**

**Appendix 1. PubMed search strategy.**

#44 Search (((((((((((((best practice*) OR guideline*) OR standard*) OR framework*) OR care pathway*) OR quality) OR "Standard of Care"[Mesh]) OR "Patient Preference"[Mesh]) OR "Patient Satisfaction"[Mesh]) OR ("Health Care Quality, Access, and Evaluation"[Mesh]))) AND (((("Health Services Accessibility"[Mesh]) OR "Delivery of Health Care, Integrated"[Mesh]) OR

"Delivery of Health Care"[Mesh]) OR (( "Health Services Administration/nursing"[Mesh] OR

"Health Services Administration/organization and administration"[Mesh] OR "Health Services Administration/supply and distribution"[Mesh] )))) AND

(("Home Care Services, Hospital-Based”[Mesh]) OR "Home Care Services"[Mesh]))

AND ((((("integrative palliative care") OR "end of life care") OR #6) OR "Palliative Care"[Mesh])

OR "Hospice Care"[Mesh]) Filters: Humans; English; French **1349**

#43 Search (((((((((((((best practice*) OR guideline*) OR standard*) OR framework*) OR care pathway*) OR quality) OR "Standard of Care"[Mesh]) OR "Patient Preference"[Mesh])

OR "Patient Satisfaction"[Mesh]) OR ("Health Care Quality, Access, and Evaluation"[Mesh]))) AND (((("Health Services Accessibility"[Mesh]) OR "Delivery of Health Care, Integrated"[Mesh])

OR "Delivery of Health Care"[Mesh]) OR (( "Health Services Administration/nursing"[Mesh] OR "Health Services Administration/organization and administration"[Mesh] OR "Health Services Administration/supply and distribution"[Mesh] ))))

AND (("Home Care Services, Hospital-Based"[Mesh]) OR "Home Care Services"[Mesh])) AND ((((("integrative palliative care") OR "end of life care") OR #6)

OR "Palliative Care"[Mesh]) OR "Hospice Care"[Mesh]) Filters: Humans; English  **1346**

#42 Search (((((((((((((best practice*) OR guideline*) OR standard*) OR framework*) OR care pathway*) OR quality) OR "Standard of Care"[Mesh]) OR "Patient Preference"[Mesh])

OR "Patient Satisfaction"[Mesh]) OR ("Health Care Quality, Access, and Evaluation"[Mesh])))

AND (((("Health Services Accessibility"[Mesh]) OR "Delivery of Health Care, Integrated"[Mesh])

OR "Delivery of Health Care"[Mesh]) OR (( "Health Services Administration/nursing"[Mesh]

OR "Health Services Administration/organization and administration"[Mesh]

OR "Health Services Administration/supply and distribution"[Mesh] ))))

AND (("Home Care Services, Hospital-Based"[Mesh]) OR "Home Care Services"[Mesh]))

AND ((((("integrative palliative care") OR "end of life care") OR #6)

OR "Palliative Care"[Mesh]) OR "Hospice Care"[Mesh]) Filters: Humans  **1540**

#41 Search (((((((((((((best practice*) OR guideline*) OR standard*) OR framework*)

OR care pathway*) OR quality) OR "Standard of Care"[Mesh]) OR "Patient Preference"[Mesh])

OR "Patient Satisfaction"[Mesh]) OR ("Health Care Quality, Access, and Evaluation"[Mesh])))

AND (((("Health Services Accessibility"[Mesh]) OR "Delivery of Health Care, Integrated"[Mesh])

OR "Delivery of Health Care"[Mesh]) OR (( "Health Services Administration/nursing"[Mesh]

OR "Health Services Administration/organization and administration"[Mesh]

OR "Health Services Administration/supply and distribution"[Mesh] ))))

AND (("Home Care Services, Hospital-Based"[Mesh]) OR "Home Care Services"[Mesh]))

AND ((((("integrative palliative care") OR "end of life care") OR #6)

OR "Palliative Care"[Mesh]) OR "Hospice Care"[Mesh]) **1573**

#40 Search (((((((((best practice*) OR guideline*) OR standard*) OR framework*)

OR care pathway*) OR quality) OR "Standard of Care"[Mesh]) OR "Patient Preference"[Mesh])

OR "Patient Satisfaction"[Mesh])

OR ("Health Care Quality, Access, and Evaluation"[Mesh]) **7213170**

#39 Search best practice*  **15214**

#38 Search guideline*  **337539**

#37 Search standard* **1488112**

#36 Search framework* **179339**

#35 Search care pathway* **2403**

#34 Search quality 866287

#33 Search "Standard of Care"[Mesh] **1883**

#31 Search "Patient Preference"[Mesh] **4444**

#29 Search "Patient Satisfaction"[Mesh]  **69584**

#27 Search "Health Care Quality, Access, and Evaluation"[Mesh]  **6032585**

#25 Search ((("Health Services Accessibility"[Mesh]) OR "Delivery of Health Care,

Integrated"[Mesh]) OR "Delivery of Health Care"[Mesh]) OR (( "Health Services Administration/nursing"[Mesh] OR "Health Services Administration/organization and administration"[Mesh] OR "Health Services Administration/supply and distribution"[Mesh])) **1131298**

#24 Search "Health Services Accessibility"[Mesh]  **91269**

#22 Search "Delivery of Health Care, Integrated"[Mesh] **9772**

#20 Search "Delivery of Health Care"[Mesh] 881255

#18 Search ( "Health Services Administration/nursing"[Mesh] OR "Health Services Administration/organization and administration"[Mesh] OR

"Health Services Administration/supply and distribution"[Mesh]) **448480**

#15 Search ("Home Care Services, Hospital-Based"[Mesh]) OR "Home Care Services"[Mesh]  **42136**

#14 Search "Home Care Services, Hospital-Based"[Mesh] **1717**

#12 Search "Home Care Services"[Mesh] 42136

#10 Search (((("integrative palliative care") OR "end of life care") OR #6)

OR "Palliative Care"[Mesh]) OR "Hospice Care"[Mesh]  **82637**

#9 Search (((("integrative palliative care") AND "end of life care")

AND "Palliative Care"[Mesh]) AND "Hospice Care"[Mesh]) AND Terminal Care"[Mesh]  **1**

#8 Search "integrative palliative care" **10**

#7 Search "end of life care" **7132**

#6 Search "Terminal Care"[Mesh] **43904**

#4 Search "Palliative Care"[Mesh] **44536**

#2 Search "Hospice Care"[Mesh] **5133**
