# Supplementary material for: Palliative care in the home: a scoping review of study quality, primary outcomes, and thematic component analysis
Source: BMC Palliat Care. 2018 Mar 7;17:41. doi: 10.1186/s12904-018-0299-z (PMC5842572; doi:10.1186/s12904-018-0299-z)
Supplement: Supplementary file 2 — Table S1. Included study characteristics. (DOCX 48 kb) [file 12904_2018_299_MOESM2_ESM.docx]

Additional file 2: Table S1. Included study characteristics.

| **First Author Country Year** | **Study Design** | **Objective** | **Primary Outcome** | **Population (n)** | **Themes** | **Quality Assessment Tool** | **Quality Assessment Outcome (Risk of Bias)** |
| --- | --- | --- | --- | --- | --- | --- | --- |
| Aabom, B. Denmark 2006[1] | Case Control | To analyze the effect of GP home visits on the granting of a terminal declaration and on place of death. | Place of death | Cancer patients (n=2025) | Individualized patient centered care | Newcastle-Ottawa Scale | Low |
| Ahlner-Elmqvist, M. Sweden 2008[2] | Cross-sectional Study | To identify differences in clinical characteristics in patients preferring advanced home care or conventional care. | Symptoms | Cancer patients (n=297) | Accessibility of healthcare Individualized patient centered care Multidisciplinary care provision | Quality Assessment Tool for Quantitative Studies | Medium |
| Aoki, N. Japan 2006[3] | Cost Analysis | To evaluate tele-palliative care to investigate its clinical and economic impact. | Cost analysis | Palliative patients (n=2) | Accessibility of healthcare Individualized patient centered care | NA | NA |
| Aoun, S. Australia 2008[4] | Qualitative | To investigate the support needs of people living alone with a terminal illness from a client perspective. | Qualitative | Palliative patients (n=11) | Accessibility of healthcare Caregiver support Individualized patient centered care | Critical Appraisal Skills Programme | Low |
| Aoun, S. Australia 2012[5] | Qualitative | To describe the experiences of terminally ill 'home alone' people using one of two models of care aimed at maintaining participant’s need for independent living. | Qualitative | Palliative patients (n=26) | Accessibility of healthcare Individualized patient centered care | Critical Appraisal Skills Programme | Medium |
| Aoun, S.M. Australia 2013[6] | Randomized Controlled Trial | To assess the feasibility of using a randomized controlled trial approach. | Symptom burden | Palliative patients (n=43) | Accessibility of healthcare Caregiver support Individualized patient centered care | Cochrane Risk of Bias Tool | Low |
| Appelin, G. Sweden 2005[7] | Qualitative | To identify the comprehensive picture of palliative care in the home, as experienced by the people involved. | Qualitative | Cancer patients, next of kin, district nurses (n=18) | Caregiver support Individualized patient centered care | Critical Appraisal Skills Programme | Medium |
| Arland, L. C United States 2013[8] | Retrospective Cohort | To evaluate an end-of-life program related to specific outcomes for children with brain tumors. | Multiple | Children with admission for pediatric brain tumors (n=166) | Individualized patient centered care Multidisciplinary care provision Quality improvement | Newcastle-Ottawa Scale | Medium |
| Bradford, N. Australia 2012[9] | Quasi-experimental | To investigate the effectiveness of a home telehealth service for pediatric palliative care consultations. | Quality of Life | Caregivers of pediatric palliative patients (n=14) | Accessibility of Healthcare Individualized patient centered care | Cochrane Risk of Bias Tool | Medium |
| Bradford, N. Australia 2014[10] | Cost Analysis | To compare the actual costs of the home telehealth program with the estimated potential costs associated with face-to-face consultations. | Cost analysis | Palliative patients (n=95) | Accessibility of Healthcare Individualized patient centered care | NA | NA |
| Brännström, M. Sweden 2014[11] | Randomized Controlled Trial | To evaluate the outcome of person-centered and integrated palliative advanced home care and heart failure care with regard to patient symptoms, health-related quality of life, and hospitalizations compared with usual care. | Multiple | Chronic heart failure palliative patients (n=72) | Individualized patient centered care Multidisciplinary care provision | Cochrane Risk of Bias Tool | Medium |
| Brink, P. Canada 2011[12] | Cross-sectional Study | To examine emergency department use by end-of-life home care clients. | Resource use | Home care clients (n=93) | Individualized patient centered care Multidisciplinary care provision | Quality Assessment Tool for Quantitative Studies | Medium |
| Brumley, R. United States 2007[13] | Randomized Controlled Trial | To determine whether and in-home palliative care intervention can improve patient satisfaction, reduce medical care costs, and increase the proportion of patients dying at home. | Multiple | Palliative patients (n=298) | Accessibility of healthcare Individualized patient centered care Multidisciplinary care provision | Cochrane Risk of Bias Tool | Low |
| Brumley, R.D. United States 2003[14] | Quasi-experimental | To evaluate the effectiveness of a palliative program for end-of-life care. | Multiple | Palliative patients (n=300) | Accessibility of healthcare Individualized patient centered care Multidisciplinary care provision | Cochrane Risk of Bias Tool | Medium |
| Cartoni, C. Italy 2007[15] | Cost Analysis | To analyze the use of resources and the costs of a program of home care. | Cost analysis | Palliative patients with hematological malignancies (n=144) | Accessibility of healthcare Individualized patient centered care Multidisciplinary care provision | NA | NA |
| Chen, C.Y. United States 2015[16] | Retrospective Cohort | To evaluate inpatient hospital utilization and adequacy of advance care planning. | Resource use | Home care clients (n=54) | Individualized patient centered care Multidisciplinary care provision | Newcastle-Ottawa Scale | Low |
| Currow, D.C. Australia 2009[17] | Retrospective cohort | To evaluate the effect of palliative home oxygen on refractory dyspnea. | Symptoms | Palliative patients with refractory dyspnea (n=413) | Accessibility of healthcare Multidisciplinary care provision | Newcastle-Ottawa Scale | Medium |
| Doolittle, G.C. United States 2000[18] | Cost Analysis | To measure costs for traditional hospice care as well as those associated with launching and operating a tele-hospice service. | Cost analysis | Palliative patients (n=1737) | Accessibility of healthcare Multidisciplinary care provision | NA | NA |
| Duggleby, W.D. Canada 2007[19] | Randomized Controlled Trial | To evaluate the effectiveness of a psychosocial supportive intervention in increasing hope and quality of life for older adult, community-living, terminally ill cancer patients. | Quality of Life | Cancer patients (n=61) | Individualized patient centered care | Cochrane Risk of Bias Tool | Medium |
| Edes, T.  United States 2006[20] | Pre-post Design | To examine the impact on hospitalization and care cost of an integrated system of end of life home and interdisciplinary care. | Multiple | Patients with congestive heart failure and chronic obstructive pulmonary disease (n=43) | Individualized patient centered care Multidisciplinary care provision | Cochrane Risk of Bias Tool | Medium |
| Enguidanos, S.M. United States 2005[21] | Quasi-experimental | To examine differences in site of death and costs of services by primary diagnosis for patients receiving home-based palliative care. | Multiple | Terminally ill patients diagnoses with cancer, congestive heart failure, or chronic obstructive pulmonary disease (n=298) | Multidisciplinary care provision | Cochrane Risk of Bias Tool | Medium |
| Fernandes, R. United States 2010[22] | Prospective Cohort | To measure symptom relief and quality of life, resource utilization, and satisfaction with home-based palliative care. | Multiple | Home care clients (n=91) | Individualized patient centered care Multidisciplinary care provision | Newcastle-Ottawa Scale | Medium |
| Grande, G.E. United Kingdom 2000[23] | Randomized Controlled Trial | To evaluate the impact of a hospital at home service on patients' quality of care, likelihood of remaining at home in their final 2 weeks of life and GP visits. | Multiple | Palliative patient caregivers, district nurses, and GPs (n=563) | Accessibility of healthcare | Cochrane Risk of Bias Tool | Low |
| Grande, G.E. United Kingdom 2004[24] | Qualitative | To identify what informal caregivers valued in the palliative support provided by GPs and district nurses by using caregivers' own descriptions of such support. | Qualitative | Bereaved caregivers (n=60) | Accessibility of healthcare Caregiver support | Critical Appraisal Skills Programme | Low |
| Groh, G. Germany 2013[25] | Quasi-experimental | To evaluate the effectiveness of one of the first specialized outpatient palliative care teams. | Satisfaction | Palliative patients and their caregivers (n=113) | Accessibility of healthcare Caregiver support | Cochrane Risk of Bias Tool | Medium |
| Guerriere, D.N. Canada 2013[26] | Prospective cohort | To measure family caregivers' satisfaction with home-based physician and nursing palliative care services. | Satisfaction | Family caregivers of palliative patients  (n=104) | Caregiver support Individualized patient centered care | Newcastle-Ottawa Scale | Medium |
| Hwang, W. Taiwan 2002[27] | Prospective Cohort | To assess the adequacy of prescribed analgesic drugs using guidelines developed by the WHO. | Symptoms | Cancer patients (n=131) | Individualized patient centered care Multidisciplinary care provision | Newcastle-Ottawa Scale | Medium |
| Jack, B.A. United Kingdom 2013[28] | Qualitative | To explore health care professionals' perspective of hospice at home service that has different components, individually tailored to meet the needs of patients. | Qualitative | Health care professionals (n=75) | Accessibility of healthcare Individualized patient centered care | Critical Appraisal Skills Programme | Medium |
| Jack, B.A. United Kingdom 2014[29] | Qualitative | To explore bereaved family caregivers' perceptions and experiences of a hospice at home service. | Qualitative | Family caregivers of palliative patients  (n=20) | Caregiver support | Critical Appraisal Skills Programme | Low |
| Kerr, C.W. United States 2014[30] | Prospective Cohort | To evaluate the clinical impact of a home-based palliative care program. | Symptoms | Home care clients (n=499) | Accessibility of healthcare Individualized patient centered care Multidisciplinary care provision | Newcastle-Ottawa Scale | Medium |
| Klinger, C.A. Canada 2011[31] | Cost Analysis | To analyze resource utilization and costs of a shared-care demonstration project. | Cost analysis | Palliative patients (n=95) | Quality improvement | NA | NA |
| Kristjanson, L.J. Australia 2004[32] | Qualitative | To develop and evaluate a community night respite palliative care service for patients and family caregivers. | Qualitative | Palliative patients (n=53) | Accessibility of healthcare Caregiver support | Critical Appraisal Skills Programme | Medium |
| Kusajima, E. Japan 2009[33] | Prospective Cohort | To clarify patients' characteristics and the level of symptom management in the transition to specialized home palliative care. | Symptoms | Cancer patients (n=100) | Individualized patient centered care | Newcastle-Ottawa Scale | Medium |
| Linderholm, M. Sweden 2010[34] | Qualitative | To explore how informal caregivers of a dying relative in palliative home care experienced their caring role and support during the patient's final illness and after death. | Qualitative | Family caregivers of palliative patients  (n=13) | Caregiver support | Critical Appraisal Skills Programme | Low |
| Lukas, L. United States 2013[35] | Pre-post Design | To evaluate a home-based, non-hospice, palliative medicine consultation practice within a fee-for-service environment. | Resource use | Patients with advance complex illness (n=369) | Individualized patient centered care | Cochrane Risk of Bias Tool | Medium |
| Maida, V. Canada 2002[36] | Retrospective Cohort | To assess factors associated with successful home death. | Place of death | Cancer patients who wished to die at home (n=402) | Accessibility of healthcare | Newcastle-Ottawa Scale | Low |
| Marshall, D. Canada 2008[37] | Qualitative | To implement and evaluate a shared model of care that aims to enhance family physician's ability to deliver quality palliative home care. | Qualitative | Family physicians (n=21) | Multidisciplinary care provision Quality improvement | Critical Appraisal Skills Programme | Medium |
| McMillan, S.C. United States 2007[38] | Randomized controlled trial | To test an intervention for hospice caregivers designed to help them better manage symptoms experienced by patients with cancer. | Symptoms | Hospice homecare patients with cancer and their caregivers (n=329) | Caregiver support Individualized patient centered care | Cochrane Risk of Bias Tool | Medium |
| Melin-Johansson, C. Sweden 2010[39] | Pre-post Design | To describe and compare quality of life before and after designation to a palliative homecare team in patients with cancer diagnoses. | Quality of Life | Cancer patients (n=63) | Accessibility of healthcare Individualized patient centered care | Cochrane Risk of Bias Tool | Medium |
| Neergaard, M.A. Denmark 2009[40] | Cross-sectional Study | To describe cancer patients in palliative care at home and examine associations between home death and GP involvement in the palliative pathway. | Place of death | Cancer patients (n=599) | Accessibility of healthcare Individualized patient centered care | Quality Assessment Tool for Quantitative Studies | Medium |
| Ornstein, K. United States 2013[41] | Prospective Cohort | To determine whether high symptom burden decreases following home-based primary and palliative care enrollment. | Symptoms | Homebound patients (n=149) | Individualized patient centered care Multidisciplinary care provision | Newcastle-Ottawa Scale | Medium |
| Pace, A. Italy 2012[42] | Cost Analysis | To evaluate the effectiveness of a home-care model of assistance in reducing the re-hospitalization rate. | Cost Analysis | Palliative patients (n=143) | Multidisciplinary care provision | NA | NA |
| Paré, G. Canada 2009[43] | Pre-post Design | To evaluate the effects of providing home care nurses with PC tablets for documentation. | Satisfaction | Home care nurses (n=7) | Individualized patient centered care Quality improvement | Cochrane Risk of Bias Tool | High |
| Riolfi, M. Italy 2013[44] | Retrospective Cohort | To assess the effectiveness of appropriate palliative home-care services in reducing hospital admissions. | Resource use | Cancer patients (n=402) | Accessibility of healthcare | Newcastle-Ottawa Scale | Low |
| Seow, H. Canada 2010[45] | Retrospective Cohort | To evaluate whether Ontario's End-of-Life Care Strategy reduced use of acute care services for palliation. | Resource use | Home care clients (n=9368) | Accessibility of healthcare Individualized patient centered care Multidisciplinary care provision | Newcastle-Ottawa Scale | Medium |
| Seow, H. Canada 2010[46] | Retrospective Cohort | To investigate whether admission time to homecare and the amount of services are associated with using acute care services at end-of-life. | Resource use | Home care clients (n=9018) | Accessibility of healthcare Individualized patient centered care | Newcastle-Ottawa Scale | Low |
| Serra-Prat, M. Spain 2001[47] | Cost Analysis | To provide a comparative assessment of the health care resources consumed during the final month of life of palliative patients. | Cost analysis | Cancer patients (n=155) | Multidisciplinary care provision | NA | NA |
| Singer, Y. Israel 2005[48] | Qualitative | To evaluate caregivers' experiences concerning the care of a terminally ill loved one at home, and to compare the death experiences of caregivers with and without access to homecare programs. | Qualitative | Caregivers of palliative patients (n=159) | Accessibility of healthcare Caregiver support Multidisciplinary care provision | Critical Appraisal Skills Programme | High |
| Tan, W.S. Singapore 2016[49] | Retrospective Cohort | To evaluate the impact of an integrated hospice home care programme on acute care service usage and on the share of home deaths. | Multiple | Cancer patients (n=914) | Accessibility of healthcare Individualized patient centered care Multidisciplinary care provision Quality Improvement | Newcastle-Ottawa Scale | Low |
| Tateno, Y. Japan 2012[50] | Pre-post Design | To evaluate the effectiveness of the clinical pathway in home palliative care on a remote island. | Symptoms | Homecare clients (n=24) | Individualized patient centered care | Cochrane Risk of Bias Tool | Medium |
| Vollenbroich, R. Germany 2012[51] | Cross-sectional Study | To evaluate the effectiveness of a specialized pediatric palliative home care team as experienced by parents and health care professionals. | Satisfaction | Parents of pediatric palliative patients (n=43) | Caregiver support Individualized patient centered care Multidisciplinary care provision | Quality Assessment Tool for Quantitative Studies | High |
| Walsh, K. United Kingdom 2007[52] | Randomized Controlled Trial | To evaluate the effectiveness of increased support for distressed, informal caregivers of patients receiving palliative care. | Caregiver distress | Informal caregivers of palliative patients (n=271) | Caregiver support Multidisciplinary care provision Quality improvement | Cochrane Risk of Bias Tool | Low |
| Wong, R.C.C. Singapore 2013[53] | Pre-post Design | To evaluate the impact of a home-based advance care programme on healthcare utilization. | Resource use | Palliative heart failure patients (n=44) | Accessibility of healthcare Multidisciplinary care provision | Cochrane Risk of Bias Tool | Medium |

**References**

1. Aabom B, Kragstrup J, Vondeling H, Bakketeig LS, Stovring H. Does persistent involvement by the GP improve palliative care at home for end-stage cancer patients? Palliat Med. 2006;20:507–12.

2. Ahlner-Elmqvist M, Jordhøy MS, Bjordal K, Jannert M, Kaasa S. Characteristics and Quality of Life of Patients Who Choose Home Care at the End of Life. J Pain Symptom Manage. 2008;36:217–27.

3. Aoki N, Ohta S, Yamamoto H, Kikuchi N, Dunn K. Triangulation analysis of tele-palliative care implementation in a rural community area in Japan. Telemed J E Health. 2006;12:655–662.

4. Aoun S, Kristjanson LJ, Oldham L, Currow D. A qualitative investigation of the palliative care needs of terminally ill people who live alone. Collegian. 2008;15:3–9.

5. Aoun S, O’Connor M, Skett K, Deas K, Smith J. Do models of care designed for terminally ill “home alone” people improve their end-of-life experience? A patient perspective: “home alone” models of care. Health Soc Care Community. 2012;20:599–606.

6. Aoun SM, O’Connor M, Breen LJ, Deas K, Skett K. Testing models of care for terminally ill people who live alone at home: is a randomised controlled trial the best approach?: Models of care for the terminally ill living alone. Health Soc Care Community. 2013;21:181–90.

7. Appelin G, Brobäck G, Berterö C. A comprehensive picture of palliative care at home from the people involved. Eur J Oncol Nurs. 2005;9:315–24.

8. Arland LC, Hendricks-Ferguson VL, Pearson J, Foreman NK, Madden JR. Development of an in-home standardized end-of-life treatment program for pediatric patients dying of brain tumors: Development of an In-Home Standardized End-of-Life Treatment Program for Pediatric Patients Dying of Brain Tumors. J Spec Pediatr Nurs. 2013;18:144–57.

9. Bradford N, Young J, Armfield NR, Bensink ME, Pedersen L -a., Herbert A, et al. A pilot study of the effectiveness of home teleconsultations in paediatric palliative care. J Telemed Telecare. 2012;18:438–42.

10. Bradford NK, Armfield NR, Young J, Smith AC. Paediatric palliative care by video consultation at home: a cost minimisation analysis. BMC Health Serv Res. 2014;14:1–9.

11. Brännström M, Boman K. Effects of person-centred and integrated chronic heart failure and palliative home care. PREFER: a randomized controlled study: PREFER: a randomized controlled study. Eur J Heart Fail. 2014;16:1142–51.

12. Brink P, Partanen L. Emergency Department Use among End-of-Life Home Care Clients. J Palliat Care. 2011;27:224–8.

13. Brumley R, Enguidanos S, Jamison P, Seitz R, Morgenstern N, Saito S, et al. Increased Satisfaction with Care and Lower Costs: Results of a Randomized Trial of In-Home Palliative Care: RANDOMIZED IN-HOME PALLIATIVE CARE TRIAL. J Am Geriatr Soc. 2007;55:993–1000.

14. Brumley RD, Enguidanos S, Cherin DA. Effectiveness of a home-based palliative care program for end-of-life. J Palliat Med. 2003;6:715–724.

15. Cartoni C, Brunetti GA, D’Elia GM, Breccia M, Niscola P, Marini MG, et al. Cost analysis of a domiciliary program of supportive and palliative care for patients with hematologic malignancies. haematologica. 2007;92:666–673.

16. Chen CY, Thorsteinsdottir B, Cha SS, Hanson GJ, Peterson SM, Rahman PA, et al. Health Care Outcomes and Advance Care Planning in Older Adults Who Receive Home-Based Palliative Care: A Pilot Cohort Study. J Palliat Med. 2015;18:38–44.

17. Currow D, Agar M, Smith J, Abernethy A. Does palliative home oxygen improve dyspnoea? A consecutive cohort study. Palliat Med. 2009;23:309–16.

18. Doolittle GC. A cost measurement study for a home-based telehospice service. J Telemed Telecare. 2000;6 suppl 1:193–195.

19. Duggleby WD, Degner L, Williams A, Wright K, Cooper D, Popkin D, et al. Living with Hope: Initial Evaluation of a Psychosocial Hope Intervention for Older Palliative Home Care Patients. J Pain Symptom Manage. 2007;33:247–57.

20. Edes TE, Lindbloom EJ, Deal JL, Madsen RW. Improving Care at Lower Cost for End-Stage Heart and Lung Disease: Integrating End of Life Planning with Home Care. Univ Mo Fam Community Med. 2006;103:146–51.

21. Enguidanos SM, Cherin D, Brumley R. Home-Based Palliative Care Study: Site of Death, and Costs of Medical Care for Patients with Congestive Heart Failure, Chronic Obstructive Pulmonary Disease, and Cancer. J Soc Work End--Life Palliat Care. 2005;1:37–56.

22. Fernandes R, Braun KL, Ozawa J, Compton M, Guzman C, Somogyi-Zalud E. Home-based palliative care services for underserved populations. J Palliat Med. 2010;13:413–419.

23. Grande GE, Todd CJ, Barclay SIG, Farquhar MC. A randomized controlled trial of a hospital at home service for the terminally ill. Palliat Med. 2000;14:375–385.

24. Grande GE, Farquhar MC, Barclay SIG, Todd CJ. Valued aspects of primary palliative care: content analysis of bereaved carers’ descriptions. Br J Gen Pract. 2004;54:772–8.

25. Groh G, Vyhnalek B, Feddersen B, Führer M, Borasio GD. Effectiveness of a Specialized Outpatient Palliative Care Service as Experienced by Patients and Caregivers. J Palliat Med. 2013;16:848–56.

26. Guerriere DN, Zagorski B, Coyte PC. Family caregiver satisfaction with home-based nursing and physician care over the palliative care trajectory: Results from a longitudinal survey questionnaire. Palliat Med. 2013;27:632–8.

27. Hwang W, Tasi Y, Chang H, Liu I, Huang C. A Prospective Study of Pain Treatment for Patients with Advanced Cancer Who Receive Hospice Home Care. Chin Med J Taipei. 2002;65:331–5.

28. Jack BA, Baldry CR, Groves KE, Whelan A, Sephton J, Gaunt K. Supporting home care for the dying: an evaluation of healthcare professionals’ perspectives of an individually tailored hospice at home service. J Clin Nurs. 2013;22:2778–86.

29. Jack BA, O’Brien MR, Scrutton J, Baldry CR, Groves KE. Supporting family carers providing end-of-life home care: a qualitative study on the impact of a hospice at home service. J Clin Nurs. 2014;24:131–40.

30. Kerr CW, Tangeman JC, Rudra CB, Grant PC, Luczkiewicz DL, Mylotte KM, et al. Clinical Impact of a Home-Based Palliative Care Program: A Hospice-Private Payer Partnership. J Pain Symptom Manage. 2014;48:883–892.e1.

31. Klinger CA, Howell D, Marshall D, Zakus D, Brazil K, Deber RB. Resource utilization and cost analyses of home-based palliative care service provision: the Niagara West End-of-Life Shared-Care Project. Palliat Med. 2011;27:115–22.

32. Kristjanson LJ, Cousins K, White K, Andrews L, Lewin G, Tinnelly C, et al. Evaluation of a night respite community palliative care service. Int J Palliat Nurs. 2004;10:84–90.

33. Kusajima E, Kawa M, Miyashita M, Kazuma K, Okabe T. Prospective Evaluation of Transition to Specialized Home Palliative Care in Japan. Am J Hosp Palliat Med. 2009;26:172–9.

34. Linderholm M, Friedrichsen M. A desire to be seen: family caregivers’ experiences of their caring role in palliative home care. Cancer Nurs. 2010;33:28–36.

35. Lukas L, Foltz C, Paxton H. Hospital Outcomes for a Home-Based Palliative Medicine Consulting Service. J Palliat Med. 2013;16:179–84.

36. Maida V. Factors that Promote Success in Home Paliative Care: a Study of a Large Suburban Palliative Care Practice. J Palliat Care. 2002;18:282–6.

37. Marshall D, Howell D, Brazil K, Howard M, Taniguchi A. Enhancing family physician capacity to deliver quality palliative home care. Can Fam Physician. 2008;54:e1-7.

38. McMillan S, Small BJ. Using the COPE Intervention for Family Caregivers to Improve Symptoms of Hospice Homeare Patients: A Clinical Trial. Oncol Nurs Forum. 2007;34:313–21.

39. Melin-Johansson C, Axelsson B, Gaston-Johansson F, Danielson E. Significant improvement in quality of life of patients with incurable cancer after designation to a palliative homecare team. Eur J Cancer Care (Engl). 2010;19:243–50.

40. Neergaard MA, Vedsted P, Olesen F, Sokolowski I, Jensen AB, Søndergaard J. Associations between home death and GP involvement in palliative cancer care. Br J Gen Pract. 2009;59:671–7.

41. Ornstein K, Wajnberg A, Kaye-Kauderer H, Winkel G, DeCherrie L, Zhang M, et al. Reduction in Symptoms for Homebound Patients Receiving Home-Based Primary and Palliative Care. J Palliat Med. 2013;16:1048–54.

42. Pace A, Di Lorenzo C, Capon A, Villani V, Benincasa D, Guariglia L, et al. Quality of Care and Rehospitalization Rate in the Last Stage of Disease in Brain Tumor Patients Assisted at Home: A Cost Effectiveness Study. J Palliat Med. 2012;15:225–7.

43. Paré G, Sicotte C, Chekli M, Jaana M, Blois CD, Bouchard M. A pre-post evaluation of a telehomecare program in oncology and palliative care. Telemed E-Health. 2009;15:154–159.

44. Riolfi M, Buja A, Zanardo C, Marangon CF, Manno P, Baldo V. Effectiveness of palliative home-care services in reducing hospital admissions and determinants of hospitalization for terminally ill patients followed up by a palliative home-care team: A retrospective cohort study. Palliat Med. 2013;28:403–11.

45. Seow H, Barbera L, Howell D, Dy SM. Did Ontario’s End-of-Life Care Strategy Reduce Acute Care Service Use? The need to use quality indicators for improvement. Healthc Q. 2010;13:93–100.

46. Seow H, Barbera L, Howell D, Dy SM. Using more end-of-life homecare services is associated with using fewer acute care services: a population-based cohort study. Med Care. 2010;48:118–124.

47. Serra-Prat M, Gallo P, Picaza JM. Home palliative care as a cost-saving alternative: evidence from Catalonia. Palliat Med. 2001;15:271–278.

48. Singer Y, Bachner YG, Shvartzman P, Carmel S. Home Death—The Caregivers’ Experiences. J Pain Symptom Manage. 2005;30:70–4.

49. Tan WS, Lee A, Yang SY, Chan S, Wu HY, Ng CWL, et al. Integrating palliative care across settings: A retrospective cohort study of a hospice home care programme for cancer patients. Palliat Med. 2016;30:634–41.

50. Tateno Y, Ishikawa S. Clinical pathways can improve the quality of pain management in home palliative care in remote locations: retrospective study on Kozu Island, Japan. Rural Remote Health. 2012;12:1992.

51. Vollenbroich R, Duroux A, Grasser M, Brandstätter M, Borasio GD, Führer M. Effectiveness of a Pediatric Palliative Home Care Team as Experienced by Parents and Health Care Professionals. J Palliat Med. 2012;15:294–300.

52. Walsh K, Jones L, Tookman A, Mason C, McLOUGHLIN J, Blizard R, et al. Reducing emotional distress in people caring for patients receiving specialist palliative care. Br J Psychiatry. 2007;190:142–147.

53. Wong RC, Tan PT, Seow YH, Aziz S, Oo N, Seow SC, et al. Home-based advance care programme is effective in reducing hospitalisations of advanced heart failure patients: a clinical and healthcare cost study. Ann Acad Med Singap. 2013;42:466–471.
